# Formulation and evaluation of nanoemulsions from *Jasminum officinale* essential oil for controlling postharvest browning and maintaining quality in jasmine (*Jasminum sambac*) flowers

**DOI:** 10.3389/fpls.2025.1541721

**Published:** 2025-02-25

**Authors:** Kittiya Yeamsuriyotai, Natthamon Pradabkun, Nutcha Manichart, Nipaporn Yonsawad, Na-monrug Khamchatra, Chamroon Laosinwattana, Montinee Teerarak, Naphat Somala

**Affiliations:** ^1^ School of Agricultural Technology, King Mongkut’s Institute of Technology Ladkrabang, Bangkok, Thailand; ^2^ Faculty of Science and Technology, Rambhai Barni Rajabhat University, Chanthaburi, Thailand

**Keywords:** antioxidant, anti-browning, freshness index, polyphenol oxidase, peroxidase, storage

## Abstract

The jasmine (*Jasminum sambac* (L.) Aiton) flower has delicate petals, resulting in rapid browning after harvest. The aim of this study was to search for an innovative postharvest treatment for delaying browning of jasmine petals using plant essential oils. *J. officinale* L. f. var. *grandiflorum* (L.) essential oil was found to reduce peroxidase activity in jasmine flower by 44.21% in the *in vitro* condition. The antioxidant activities and chemical composition of *J. officinale* essential oil were subsequently characterized. The essential oil exhibited the ability to scavenge 2,2-diphenyl-1-picrylhydrazil (DPPH) radicals with a 50% inhibition (EC_50_) value of 6.72 ± 0.89 mg/mL, a chelating effect with EC_50_ value of 7.42 ± 1.59 mg/mL, and reducing power with EC_0.5_ value of 14.89 ± 0.73 mg/mL. GC-MS analysis detected 29 compounds in the oil, with benzyl alcohol (20.68%) and benzyl acetate (19.87%) predominating. As plant essential oils have restricted water solubility, an oil-in-water emulsion was formulated using a spontaneous emulsification method. The resulting *J. officinale* essential oil naonoemulsion (JEN) had an oil droplet size of 70.2 ± 0.39 nm and a narrow polydispersity index. *In vivo* testing confirmed the inhibitory effects of JEN on jasmine flower browning and relevant enzyme activities. Jasmine flowers were soaked in various concentrations of JEN for 5 min, packed in polyethylene plastic bags, and stored in a refrigerator at 10 ± 3°C with relative humidity 66 ± 5%. Flowers treated with 1 and 2 mg/mL JEN showed effective delay of petal browning and maintained good quality with minimum flower opening index, high freshness score, and high color retention index. JEN treatment also reduced phenylalanine ammonia lyase (PAL), polyphenol oxidase (PPO), and peroxidase (POD) activities, indicating postponement of the browning process. In addition, scanning electron microscopy micrographs of treated flower epidermis cells revealed delayed cell wall collapse, indicating retention of intact cells. Taken together, these results support JEN as a potential preventative of enzymatic browning and hence petal browning in jasmine flower.

## 1 Introduction

Jasmine, a member of the Oleaceae family, is a common fragrant plant with sweet-scented flowers. The most well-known species of jasmine is *Jasminum sambac* (L.) Aiton, also known as the Arabian jasmine or jasmine flower. In several Asian countries, the jasmine flower is regarded as a propitious symbol in religious ceremonies and has been integrated into local customs and traditions. In Thailand, this flower is consumed domestically, is in demand in the domestic market throughout the year, and is popularly demanded for a traditional Mother’s Day, religious holidays, and the Songkran festival. However, jasmine flowers are delicate and highly perishable in nature with petals turning brown by the second day after harvesting (Lavanya et al., 2016; Ali et al., 2024). The primary cause of brown-colored pigment in fresh-cut products and flowers is enzymatic activity, including polyphenol oxidase (PPO) and peroxidase (POD), that contributes to phenolic oxidation to form dark-colored compounds. The centrality of this enzymatic browning reaction in regulating brown-colored pigment within petal tissues has been established in cut anthurium (Aghdam et al., 2019) and narcissus (Golrokh and Somayeh, 2023) flowers. In addition, numerous studies in a variety of cut flowers have reported that PPO and POD activity increases prior to or during petal browning, and those methods targeted at reducing browning often lower PPO and POD activities. For example, the browning and quality deterioration of *Narcissus tazetta* cv. Shahla−e−Shiraz flowers are controlled by γ−aminobutyric acid (Golrokh and Somayeh, 2023), maintains the membrane stability index and inhibits PPO and POD. In addition to PPO and POD, the enzyme phenylalanine ammonia lyase (PAL) indirectly contributes to browning by providing the substrate for the synthesis of phenolic compounds. PAL activity increases prior to or during tissue browning, and techniques aimed at minimizing browning often lower PAL activity, as has been demonstrated in fresh-cut lettuce (Chen et al., 2017) and fresh-cut taro slices (Wang et al., 2022).

Essential oils are being increasingly recognized as natural antioxidant agents, which is a valuable alternative for the preservation of agricultural produce. Antioxidant agents can also be used to inhibit or slow down oxidation and enzymatic browning of fruit and vegetable (Piva et al., 2017). Extensive research has also investigated the use of plant essential oils as anti-discoloration agents to prevent browning, for example in button mushrooms (Gao et al., 2014), fresh-cut lettuce (Chen et al., 2017), and cloudy apple juice (Xu et al., 2020). Notably, the utilization of free essential oils may be impeded by several significant factors, including their low water solubility, low stability, and susceptibility to degradation by volatilization and/or oxidation. Micro- and nanoemulsions can serve as promising delivery systems for essential oils and their bioactive constituents, typically incorporating carrier materials such as surfactant solutions, including Surfynol, Tween, Span, or lecithin (Cortés et al., 2021; Yammine et al., 2024). Compared to free essential oils or microemulsions, nanoemulsions offer the advantages of smaller droplet size, high solubilization capacity, enhanced stability, and higher antimicrobial and antioxidant activities (Salvia-Trujillo et al., 2015; Lou et al., 2017; Sundararajan et al., 2018). Nanoemulsification markedly improved the antioxidant efficacy of essential oils relative to pure essential oil (Lou et al., 2017). Thus, nanoemulsions are good candidate formulations for delivering plant essential oils that can serve as antioxidants, and anti-browning agents.

A number of chemicals have been investigated for their application to extend storage life and decrease browning caused by enzymes, including in jasmine flowers by dipping in boric acid (Boonprasert et al., 2018) and calcium carbonate solution (Sriwichan et al., 2016). As far as we know, no information is available on the response of jasmine flowers to postharvest essential oil application. The aim of this study was investigation the ability of flower essential oils in browning- relevant enzyme inactivation of jasmine flowers. An effective flower essential oil was analyzed the chemical composition and determined its antioxidant capacity. This study also reports easy methods for preparation of nanoemulsions using surfactants and the characteristics of nanoemulsion in terms of droplet size and size distribution. Nanoemulsion has emerged to enhance essential oil efficacy along with targeted delivery. Finally, given the importance of jasmine petal browning in postharvest flower quality, the effect of nanoemulsion treatment on jasmine browning and quality over 12-day cold storage was investigated.

## 2 Materials and methods

### 2.1 Chemicals

Nonionic surfactants including Tween 80, Span 20, Span 80, Span 83, and Span 85 were purchased from Sigma-Aldrich (Taufkirchen, Germany). Ascorbic acid, catechol, boric acid, 2,2-diphenyl-1-picrylhydrazil (DPPH), ethanol (≥99.8%), ferrozine, guaiacol, L-phenylalanine, polyvinylpolypyrrolidone (PVPP) and potassium ferricyanide and trichloroacetic acid were purchased from Sigma-Aldrich (Taufkirchen, Germany). Disodium hydrogen phosphate, ferric chloride, ferrous sulphate and potassium dihydrogen phosphate were purchased from Ajax Finechem (New South Wales, Australia). Hydrochloric acid and hydrogen peroxide were purchased from Merck (Darmstadt, Germany).

### 2.2 Plant material and plant essential oils

Jasmine (*Jasminum sambac* (L.) Aiton cv. Ratsburana) flowers were produced at a commercial grower’s field in Phichit province, Thailand. The flower buds were harvested by hand during the morning, pre-cooled by cold water immersion (0-4°CC) for 20 min, packed in polythene bags, stored in a foam box with ice, and transported to the laboratory as soon as possible. Flower bud samples of uniform size with milky white color and absence of mechanical injury were used for the study.

Plant essential oils from *J. sambac* (L.) Aiton, *J. officinale* L. f. var. *grandiflorum* (L.) Kob., *Gardenia augusta* (L.) Merr., *Rosa bourboniana* L. (Edward rose), and *Tagetes patula* L. were purchased from the Chemipan Corporation Co., Ltd (Bangkok, Thailand).

### 2.3 Identification of compounds in *J. officinale* essential oil

The solvent extraction method was employed to extract the volatile oil from flowers of *J. officinale*. The components of *J. officinale* essential oil were identified by GC-MS analysis. Gas chromatography-mass spectrometry (Scion 436-GC MS/MS Triple Quad, Bruker, USA) utilized a HP-5MS column (30 m length, 0.25 µm film thickness, 0.25 mm internal diameter). The injector temperature was 250°C and the split ratio 100:1. The temperature was programmed as follows: initial temperature 50°C for 2 min, ramp 20°C per minute to 220°C, and a final hold at 220°C for 18 min. The mass detector conditions were: transfer line temperature 250°C and ion source temperature 230°C; electron ionization at 70 eV with a scan range from 30 - 500 amu. Compounds were identified by comparison of their mass spectra to the mass spectral library of W10N14R.lib.

### 2.4 Determination of *J. officinale* essential oil antioxidant capacities

Antioxidant activity of the *J. officinale* essential oil in ethanol was assessed in terms of diphenyl-2-picrylhydrazyl (DPPH) radical scavenging activity and ferrous ion chelating ability following the methods of Arteaga et al. (2012) and Singh and Rajini (2004), respectively. Reducing power was also assessed according to Thaipong et al. (2006). Linear regression analysis was used to determine the sample concentrations required to reduce 50% of DPPH radicals (EC_50_), chelate 50% of Fe^2+^ ions (EC_50_), and obtain 0.5 absorbance units (A.U.), referred to as the EC_0.5_.

### 2.5 *In vitro* inhibition of browning-relevant enzymes in the presence of flower essential oil

For the determination of phenylalanine ammonia lyase (PAL, EC 4.3.1.5) activity, 45 grams of jasmine flowers were extracted with 150 mL of 50 mM borate buffer (pH 8.5) and 35 μL of 5 mM 2-mercaptoethanol containing 25 g/L PVPP using a commercial electric blender (Electrolux, Sweden). The homogenate was centrifuged at 6000×*g* for 20 min at 4°C and the supernatant collected. PAL activity was determined using the procedure described by Huang et al. (2020). The reaction mixture was prepared by adding 700 μL of 100 mM L-phenylalanine, 3 mL of borate buffer (pH 8.5), and 3 mL treatment solution (10 mg/mL flower essential oil, 100 mM ascorbic acid, or distilled water) to 300 μL of the supernatant; this mixture was then incubated at 40°C for 1 h. The reaction was finally terminated by the addition of 100 μL of 5 mM HCl, and enzyme activity was measured at an absorbance of 290 nm.

For the determination of polyphenol oxidase (PPO, EC 1.14.18.1) and peroxidase (POD, EC 1.11.1.7) activities, 45 grams of jasmine flowers were similarly extracted with 150 mL of 0.05 M phosphate buffer (pH 7.0) containing 25 g/L PVPP using an electric commercial blender. The homogenate was centrifuged using an MPW-260R (MPW, Poland) at 6000×*g* for 20 min at 4°C, and the supernatant was collected.

PPO activity was assayed using the procedure described by Chen et al. (2017). The reaction mixture consisted of 3 ml of 0.5 M catechol solution in 0.1 M phosphate buffer (pH 7.0), 0.1 mL treatment solution (10 mg/mL flower essential oil, 100 mM ascorbic acid, or distilled water), and 0.2 mL crude enzyme solution. Enzyme activity was measured at an absorbance of 420 nm with a VIS Spectrophotometer (Spectronic™ GENESYS 20 spectrophotometer, Thermo Fisher Scientific, USA).

POD activity was assayed using the procedure described by Seyyed and Shahram (2011). The mixture consisted of 0.1 mL of 0.4% guaiacol, 0.1 mL of 0.46% hydrogen peroxide, 2 mL of 0.05 M phosphate buffer, 0.1 mL treatment solution (10 mg/mL flower essential oil, 100 mM ascorbic acid, or distilled water), and 1 mL crude enzyme solution. Enzyme activity was measured at an absorbance of 470 nm.

PAL activity was expressed as units per minute per gram of fresh weight (U/min/g (FW)) and PPO and POD activities as U/min/g (FW).

### 2.6 Formulation of a *J. officinale* essential oil nanoemulsion and determination of oil droplet size and polydispersity index

Briefly, the nonionic surfactant Tween 80 was mixed with the co-surfactant Span 20, Span 80, Span 83, or Span 85 in ratios of 1:0.5, 1:1, 1:1.5, 1:2, 1:2.5, 1:3.5, and 1:4 (w/w). Then, the *J. officinale* essential oil was combined with each surfactant/co-surfactant mixture by magnetic stirring at a weight ratio of 3:1. Each mixture was then titrated dropwise into a conical flask containing a calculated amount of distilled water sufficient to make a 50 mg/mL essential oil emulsion. The emulsions were maintained in laboratory conditions for 30 days to assess their stability and checked periodically for phase separation. Only stable and milky-white emulsions were selected for measurement of the oil droplet size distribution and polydispersity index (PDI) by means of light scattering with a NanoPlus Zeta/Nano Particle Analyzer (Particulate Systems, USA).

### 2.7 Application of the *J. officinale* essential oil nanoemulsion to jasmine flower

#### 2.7.1 Preliminary screening

A stock solution of the *J. officinale* essential oil nanoemulsion (JEN, 50 mg/mL) was prepared by combining 3 g of pure *J. officinale* essential oil, 1 g of a mixture of Tween 80 and Span 85 (1:4 w/w ratio), and the appropriate amount of distilled water. This stock solution was then diluted with distillated water to produce a range of dilutions (0.01, 0.05, 0.1, 0.5, 1, and 2 mg/mL) for bioefficacy assay using postharvest jasmine flowers. Fifty flower buds of the Ratsburana jasmine variety were soaked in diluted JEN solution or distilled water for 5 min. Then, the treated samples were taken out, air-dried, packed in a polyethylene plastic bag, and stored in a refrigerator at 10 ± 3°C with relative humidity 66 ± 5%. Three replicates per treatment were sampled on days 3, 6, and 9 and color retention was assessed according to a 4-point scale where 4 = milk-white, 3 = creamy or yellowish, 2 = 1-50% brown, and 1 = 51-100% brown. The color retention index was computed using Equation 1.


(1) $$\begin{array}{c} \text{Color retention index}(\%)\\ =\frac{\sum \left(\text{score value}\times \text{flower number with given score}\right)\times 100}{4\times \text{total of number of flowers evaluated}} \end{array}$$

#### 2.7.2 Effect of JEN treatment on the browning of jasmine flowers during storage

Fifty flower buds of the Ratsburana jasmine variety were divided into four groups and soaked in 0, 0.5, 1, or 2 mg/mL JEN for 5 min. Then, the treated samples were taken out, air-dried, packed in a polyethylene plastic bag, and stored in a refrigerator at 10 ± 3°C with relative humidity 66 ± 5%. Three replicates from each treatment group were taken for evaluation at 3-day intervals for up to 12 days of storage.

##### 2.7.2.1 Quality evaluation

Weight loss was detected as the difference between weight before storage and weight after storage divided by the weight before storage. The value was expressed as a relative percentage.

Flower opening score was judged on a scale of 0 to 3 in which 0 = closed bud, 1 = partially opened, 2 = half opened, and 3 = fully opened. The flower opening index was computed using Equation 2.


(2) $$\begin{array}{c} \text{Flower opening index}(\%)\\ =\frac{\sum \left(\text{score value}\times \text{flower number with given score}\right)\times 100}{3\times \text{total of number of flowers evaluated}} \end{array}$$

Freshness index was determined from fifty flower buds. The criteria for judging freshness were based on flower development, namely bud opening, and petal browning using a score from 9 to 1 in which 9 = all buds turgid, white; 8 = partial to half open flowers, white; 7 = half to fully open flowers, white; 6 = partial to half open flowers, cream or yellowish; 5 = half to fully open flowers, cream or yellowish; 4 = partial to half open flowers, 25 to 50% brown; 3 = half to fully open flowers, 25 to 50% brown; 2 = partial to half open flowers, 75 to 100% brown; and 1 = half to fully open flowers, 75 to 100% brown. The freshness index was computed using Equation 3.


(3) $$\text{Freshness}\;\text{index}(\%)=\frac{\sum \left(\text{score value}\times \text{flower number with given score}\right)\times 100}{9\times \text{total of number of flowers evaluated}}$$

##### 2.7.2.2 Assaying PAL, PPO, and POD activities in jasmine flowers during storage

The enzyme assay methods were similar to the procedures described above, except that crude enzyme extract solutions added into the substrate buffer solution were prepared from treated jasmine flowers stored for 0, 3, 6, 9, or 12 days.

##### 2.7.2.3 Observation by scanning electron microscopy

To examine the adaxial epidermis of the petals, fresh petals from treated flowers stored for six days were rinsed with distilled water, and a square sample of 5 mm × 5 mm was excised and immersed overnight in a 2.5% (v/v) glutaraldehyde solution at 4°C. Subsequently, the petal samples were dehydrated in a graded ethanol series, then placed on pin stubs with carbon tape and sputter coated with gold. Finally, the adaxial surfaces of all samples were analyzed using a scanning electron microscope (JEOL InTouchScope™, JSM-IT500HR, JED-2300, Japan).

### 2.8 Statistical analysis

All experiments in this study were established according to a complete randomized design (CRD) with three replicates. Analysis of variance was applied using SPSS. When the ANOVA showed significant differences at *p* < 0.05, Tukey’s test was applied to compare the mean values.

## 3 Results

### 3.1 *In vitro* inhibition of PAL, PPO, and POD by flower essential oils

As presented in **Table 1**, the flower essential oils under investigation did not inhibit PAL and PPO activities. Inhibition of PAL and POD activities was provided by 100 mM ascorbic acid (as positive control). Ascorbic acid showed the greatest suppression of POD activity. In flower essential oil treatment, *J. officinale* essential oil highly inhibited POD activity, followed by *R. bourboniana* essential oil, *J. sambac* essential oil and *G. augusta* essential oil. Application of *T. patula* essential oil had no effect on POD activity. Thus, the *J. officinale* essential oil exerted the greatest inhibitory effect on POD activity in the *in vitro* condition.

**Table 1:** PAL, PPO, and POD content and of jasmine flowers in the presence of flower essential oils (10 mg/mL) and ascorbic acid as antioxidant. Numbers in parentheses indicate percentage of *in vitro* enzyme activity reduction.

Flower essential oil	PAL (U/g/hr)	PPO (U/g/min)	POD (U/g/min)
Distilled water	1534.52 (0)a	165.88 (0)b	66.26 (0)e
100 mM ascorbic acid	1469.33 (4.26)a	134.17 (19.11)a	15.59 (76.47)a
*Jasminum sambac*	2136.90 (0)a	186.32 (0)b	57.81(12.74)cd
*J. officinale*	2929.78 (0)a	211.21 (0)b	36.96 (44.21)b
*Gardenia augusta*	3174.48 (0)a	184.40 (0)b	60.37 (8.86)d
*Tagetes patula*	2373.70 (0)a	274.52 (0)b	94.63 (0)e
*Rosa bourboniana*	1801.46 (0)a	193.78 (0)b	56.00 (15.46)c

Means with different letters are significantly different at 5% probability (Tukey’s test).

### 3.2 Chemical composition and antioxidant capacity of *J. officinale* essential oil

**Figure 1:**
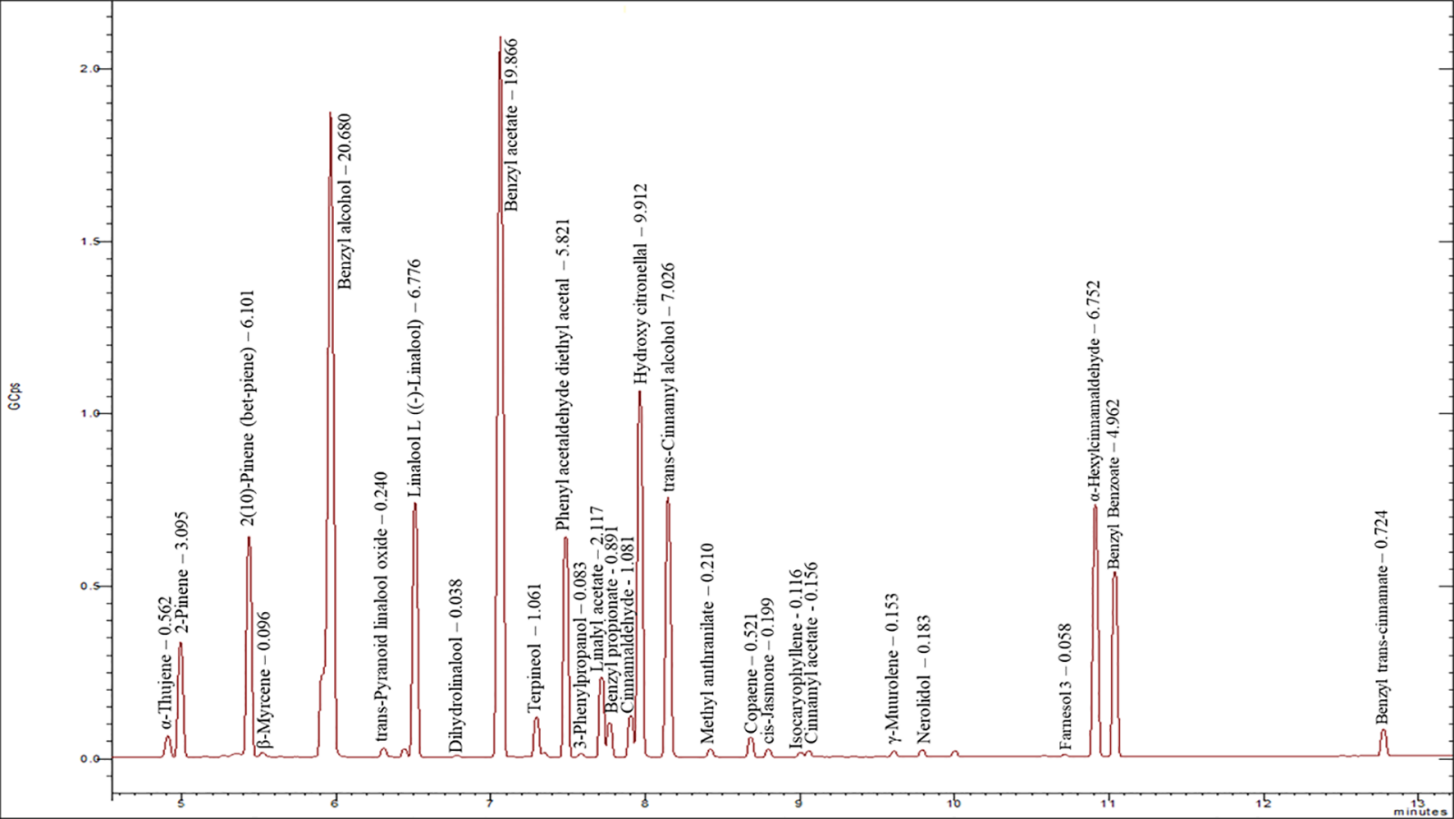
GC-MS chromatogram of essential oil of the flowers of *Jasminum officinale* showing the separation of chemical components.

The total ion chromatogram of the *J. officinale* essential oil is presented in **Figure 1**; a total of 29 compounds were identified, representing 99.52% of the total weight (**Table 2**). The major volatile components with concentrations higher than 5.0%, calculated as percent relative to total area, were benzyl alcohol (20.68%), benzyl acetate (19.87%), hydroxy citronellal (9.91%), trans-cinnamyl alcohol (7.03%), 2(10)-pinene (6.10%), linalool L (6.78%), α-hexylcinnamaldehyde (6.75%), and phenyl acetaldehyde diethyl acetal (5.82%). Additional volatiles present in medium concentrations (1.0-5.0%) were benzyl benzoate (4.96%), 2-pinene (3.10%), linalyl acetate (2.12%), cinnamaldehyde, (E)- (1.08%), and terpineol (1.06%). Many other components occurred in minor amounts (lower than 1.00%).

**Table 2:** Constituent compounds and antioxidant capacities of *J. officinale* essential oil.

No.	Peak name	Retention time	% of total
1	α-Thujene	4.911	0.562
2	2-Pinene (ALPHA-PINENE)	4.994	3.095
3	Benzaldehyde	5.275	0.043
4	2(10)-Pinene (bet-piene)	5.437	6.101
5	β-Myrcene	5.520	0.096
6	Benzyl alcohol	5.963	20.680
7	trans-Pyranoid linalool oxide	6.306	0.240
8	Linalool L ((-)-Linalool)	6.509	6.776
9	Dihydrolinalool	6.775	0.038
10	Benzyl acetate	7.060	19.866
11	Terpineol (alpha-Terpineol)	7.296	1.061
12	Phenyl acetaldehyde diethyl acetal	7.486	5.821
13	3-Phenylpropanol	7.586	0.083
14	Linalyl acetate	7.717	2.117
15	Benzyl propionate	7.769	0.891
16	Cinnamaldehyde, (E)-	7.907	1.081
17	Hydroxy citronellal	7.964	9.912
18	trans-Cinnamyl alcohol	8.146	7.026
19	Methyl anthranilate	8.424	0.210
20	Copaene	8.683	0.521
21	cis-Jasmone	8.794	0.199
22	Isocaryophyllene	9.003	0.116
23	Cinnamyl acetate	9.061	0.156
24	γ-Muurolene	9.611	0.153
25	Nerolidol	9.794	0.183
26	Farnesol 3	10.715	0.058
27	α-Hexylcinnamaldehyde	10.911	6.752
28	Benzyl Benzoate	11.038	4.962
29	Benzyl trans-cinnamate	12.776	0.724
Total identified (%)			99.52
Antioxidant capacity			
^1/^EC_50_ of DPPH free radical scavenging activity (mg/mL)			6.72 ± 0.89
^2/^EC_50_ of metal chelator (mg/mL)			7.42 ± 1.59
^3/^EC_0.5_ of reducing power (A.U.)			14.89 ± 0.73

^1^,^2/^Concentration (mg/mL) required to reduce 50% of DPPH radicals and to chelate 50% of Fe^2+^ ions, respectively.

^3/^Concentration (mg/mL) required to obtain 0.5 A.U.

The essential oil of flowers of *J. officinale* was subjected to screening for its possible antioxidant activities by three methods, namely, DPPH free radical scavenging activity, metal chelate effect and reducing power assay. The free-radical scavenging properties of the *J. officinale* essential oil are presented in **Table 2**. Plant essential oils are classified as natural antioxidants due to their capacity to reduce and/or prevent the formation of free radicals. According to the EC_50_ values presented in **Table 2**, a lower EC_50_ value indicates greater antioxidant activity. The EC_50_ values obtained were 6.72 ± 0.89 mg/mL for DPPH radical reduction and 7.42 ± 1.59 mg/mL for Fe^2+^ ion chelation. Performing both tests provides that the essential oil exhibits effective scavenging of DPPH radicals and good chelating power. With regard to reducing power, the concentration of essential oil required to obtain 0.5 A.U. was 14.89 ± 0.73 mg/mL.

### 3.3 Characterization of *J. officinale* essential oil in emulsion

**Figure 2:**
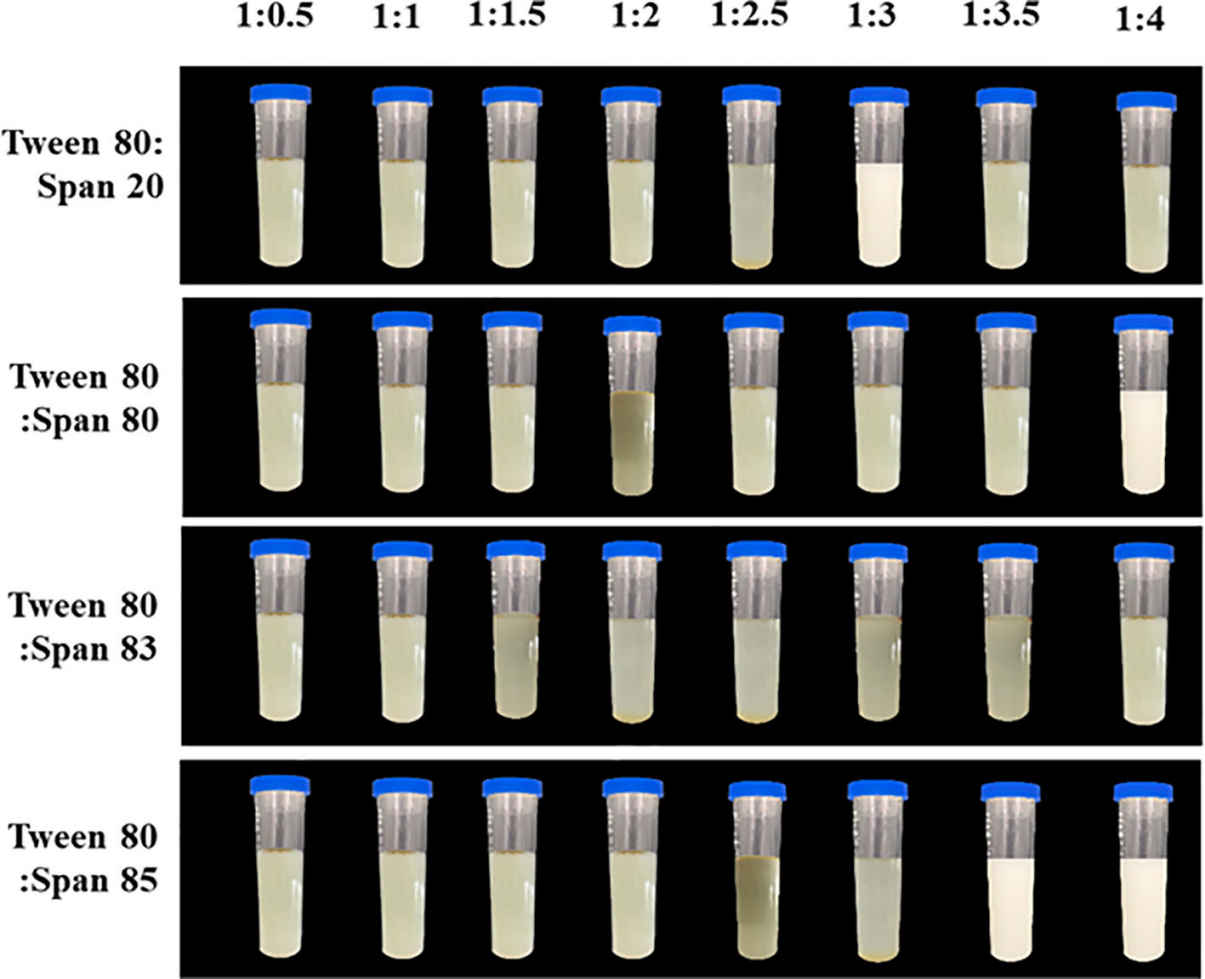
Images demonstrating the physical stability of *J. officinale* essential oil emulsion prepared by combining with surfactant Tween 80 and co-surfactant Span 20, Span 80, Span 83, or Span 85 at various ratios after 30 days storage at room temperature.

Overall, emulsions composed of *J. officinale* essential oil and high mass ratios of Tween 80 combined with Span 20, Span 80, or Span 85 exhibited greater stability than those formulated with low surfactant mass ratios. **Figure 2** illustrates the significant instability of emulsions formulated from Tween 80 and Span 83 with 5% oil content, leading to phase separation, sedimentation, or creaming at the sample surface. In contrast, mixtures of Tween 80 with Span 20, Span 80, or Span 85 in the ratios of 1:3, 1:4, and 1:3.5 enhanced emulsion stability over a 30-day storage period, with the solutions appearing visually similar to milk (**Figure 2**). The droplet sizes recorded for these stable emulsions were microscale, at 261.2, 225.8, and 139.8 nm, respectively. Based on these results, a final emulsion was formed as an aqueous solution containing 3 g of *J. officinale* essential oil and 1 g of a mixture of Tween 80 (surfactant) and Span 85 (co-surfactant) in a weight ratio of 1:4. This nanoemulsion had a nanoscale droplet size of 70.2 ± 0.39 nm. Where the microemulsions displayed polydispersity indexes of 0.261 to 0.312, the nanoemulsion demonstrated a narrower polydispersity index of 0.191 (**Table 3**).

**Table 3:** Particle size and polydispersity index of *J. officinale* essential oil emulsions.

*Jasminum officinale* emulsion formula (oil:surfactant, w/w)	Oil particle size (nm)	Polydispersity index
Oil: Tween 80 + Span 20 (1:3)	261.2 ± 2.36	0.308 ± 0.012
Oil: Tween 80 + Span 80 (1:4)	225.8 ± 2.67	0.261 ± 0.006
Oil: Tween 80 + Span 85 (1:3.5)	139.8 ± 0.80	0.312 ± 0.003
Oil: Tween 80 + Span 85 (1:4)	70.2 ± 0.39	0.191 ± 0.005

### 3.4 Application of *J. officinale* essential oil to jasmine flowers

#### 3.4.1 Preliminary screening of JEN soaking solution effectiveness

**Figure 3:**
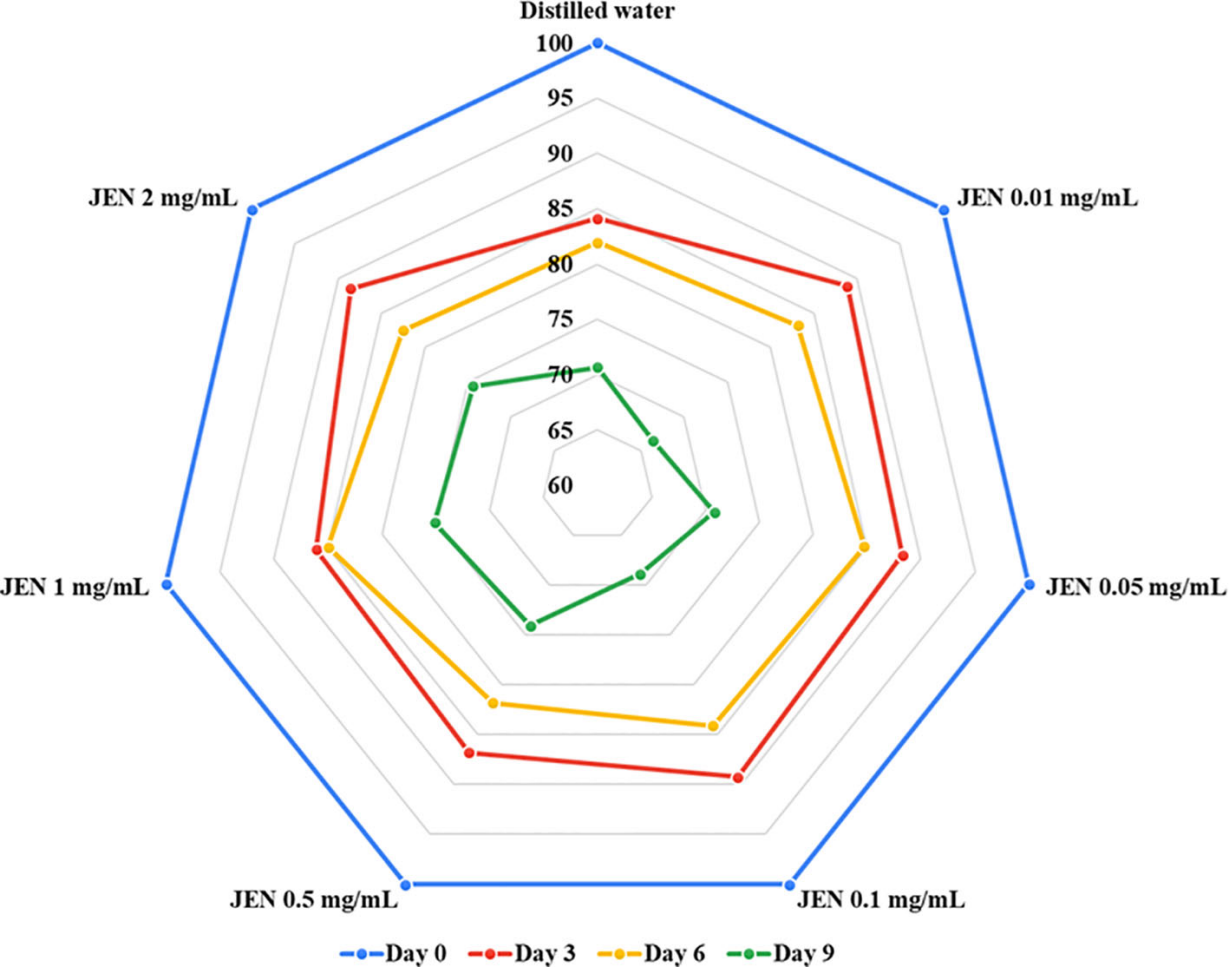
Assessment of *J. officinale* essential oil nanoemulsion (JEN) at different concentrations on the color retention index (scale: 4 = milk-white; 3 = creamy; 2 = 1-50% brown; 1 = 51-100% brown) of jasmine flowers after 9 days of storage at 10± 3°C.

*In vitro* investigations indicated the essential oil of *J. officianale* to have a significant inhibitory effect on POD activity (**Table 1**). According to **Table 2**, the antioxidant power of *J. officinale* essential oil (**Table 2**), which refers to the essential oil ability to protect cells from free radical damage through multiple processes, may directly or indirectly sequester damaging free radicals, preventing jasmine flower browning. Subsequently, preliminary experiments testing the ability of JEN to control flower browning and quality were conducted, with different concentrations of JEN applied to jasmine flowers during storage; the results are presented in **Figure 3**. Application of JEN at 0.5, 1, and 2 mg/mL delayed the appearance of browning on petals, as evidenced by color retention index values. Both the control treatment and low concentrations of JEN resulted in flowers with brown color on day 9.

#### 3.4.2 Main study of postharvest jasmine flowers

JEN treatment concentrations of 0.5, 1, and 2 mg/mL were chosen for further postharvest treatment assays to obtain more detailed insights into their effects on jasmine flowers.

##### 3.4.2.1Weight loss

**Figure 4:**
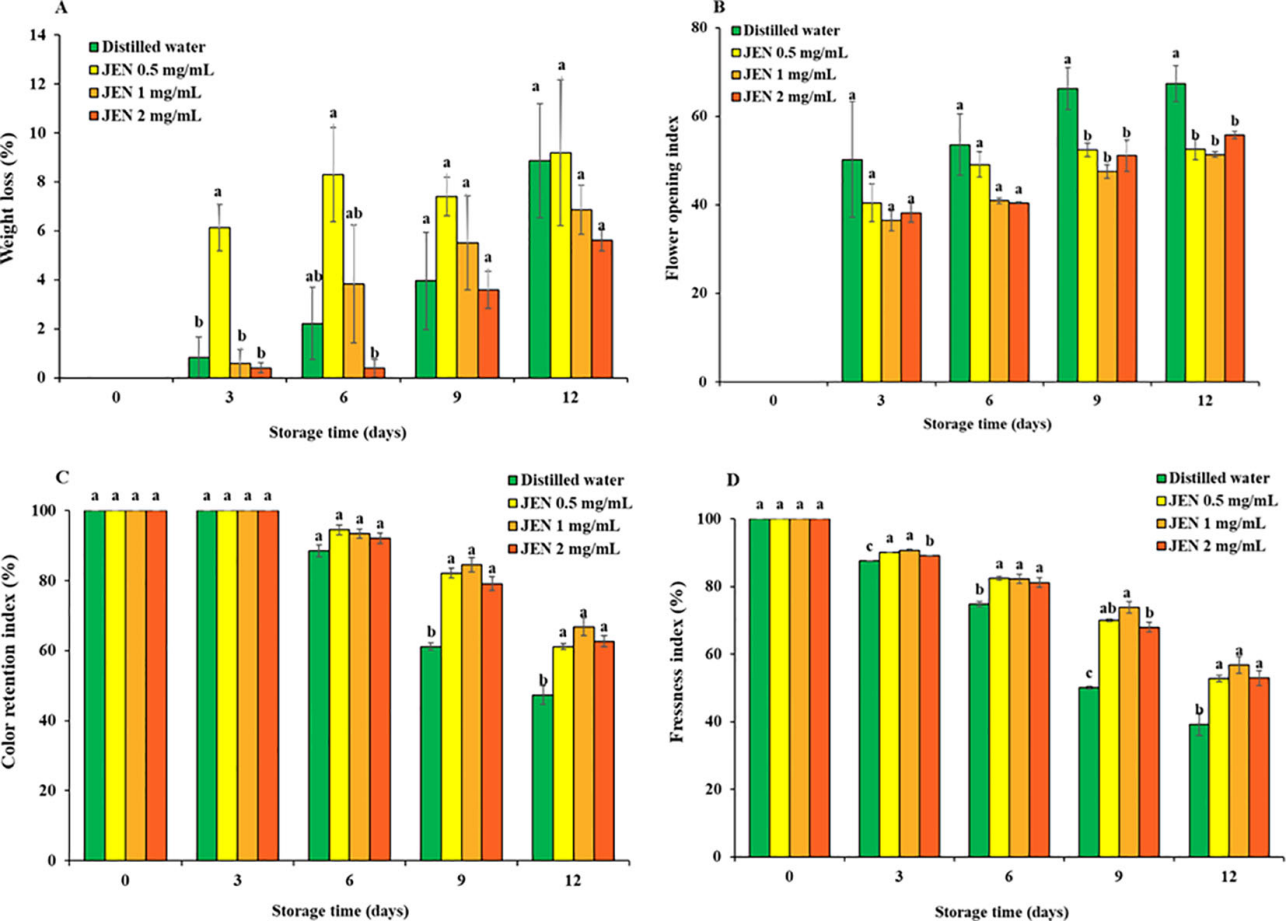
Weight loss **(A)**, flower opening index **(B)**, color retention index **(C)**, and freshness index **(D)** of jasmine flowers during storage as affect by postharvest *(J) officinale* nanoemulsion (JEN) dipping. Data are means ± SE (vertical bars). Different letters over a column cluster indicate significant differences (*p* < 0.05) among treatments according to Tukey’s test for the given timepoint.

Weight loss from jasmine flowers rose gradually as the storage period progressed in all treatments, as shown in **Figure 4A**. An initial rapid increase in weight loss during the first 3 days of storage was observed for the 0.5 mg/mL JEN treatment. Thereafter, the flowers lost fresh weight continuously, with approximately the same effect as distilled water. At the end of storage, weight loss values for the control and 0.5, 1, and 2 mg/mL JEN treatments were 8.87%, 9.18%, 6.86%, and 5.62%, respectively.

##### 3.4.2.2 Flower opening

In untreated jasmine flowers, the flower opening index continuously increased from 0% on day 0 to 67.34% on day 12, the end of the experiment. The average index of JEN-treated flowers after 0, 3, 6, 9, and 12 days of storage was 0%, 38.37%, 43.48%, 50.37%, and 53.23%, respectively. At 9 and 12 days of storage, the JEN-treated flowers exhibited a significantly lower flower opening index than the control (**Figure 4B**). Thus, the essential oil considerably postponed flower opening.

##### 3.4.2.3 Color retention

**Figure 5:**
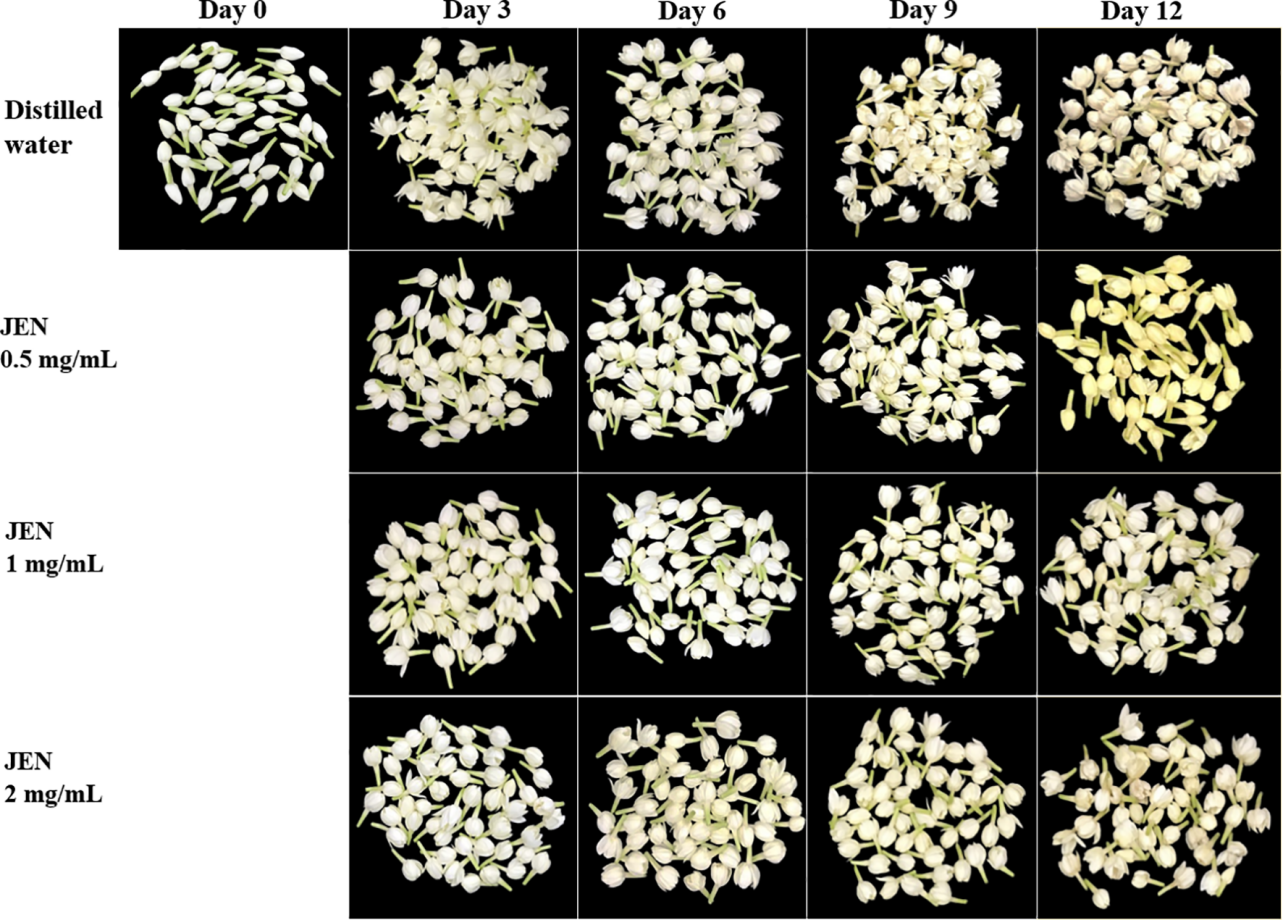
Anti-browning effect of *J. officinale* nanoemulsion (JEN) treatment on the browning of jasmine flowers during storage at 10 ± 2°C for 12 days.

In all jasmine flowers, a creamy or yellowish petal color emerged on day 3. However, flowers treated with JEN demonstrated a substantially higher color retention index than the control from day 3 to day 12. After 3, 6, 9 and 12 days of storage, the index of untreated flowers decreased to 87.56%, 74.74%, 50.07%, and finally 39.62%, while the corresponding average index value of JEN-treated flowers was 89.93%, 81.95%, 70.57%, and 53.14%, respectively (**Figure 4C**). Browing of jasmine flowers was delayed by higher concentrations of JEN (1 and 2 mg/mL), whereas both the control and the lowest concentration of JEN showed early petal browning with more loss of visual quality (**Figure 5**).

##### 3.4.2.4 Freshness index

In all jasmine flowers, petal freshness index initially decreased after 3 days of storage. On day 6, untreated flowers had a freshness index of 88.50%, while JEN-treated flowers showed higher values (>92%). On days 9 and 12, JEN treatment resulted in substantially higher freshness index values compared to untreated flowers (**Figure 4D**).

##### 3.4.2.5 Activities of PAL, PPO, and POD enzymes

**Figure 6:**
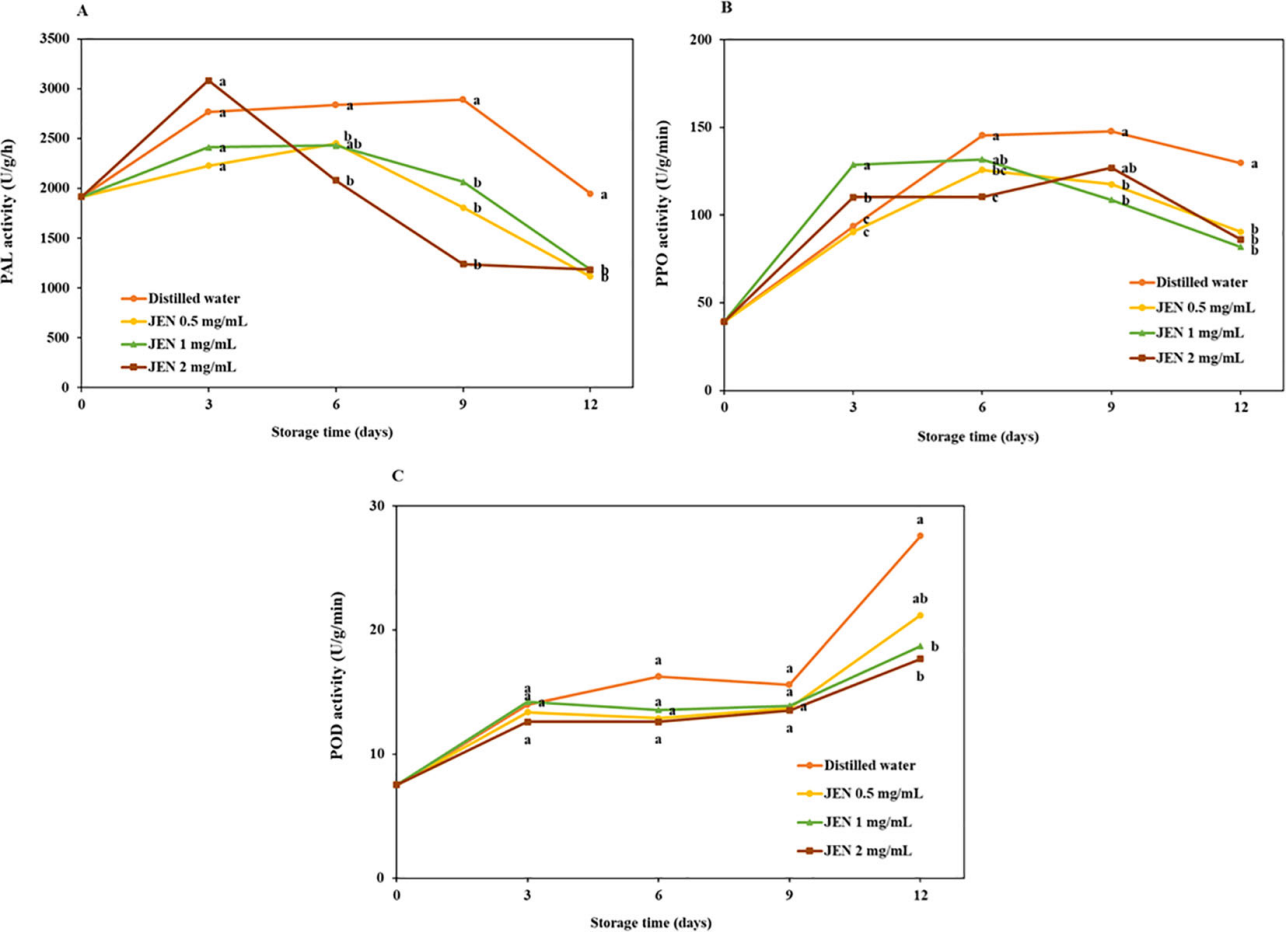
Activities of PAL **(A)**, PPO **(B)**, and POD **(C)** of jasmine flowers during storage as affect by postharvest (*J*) *officinale* nanoemulsion (JEN) dipping. Different letters indicate significant differences (*p* < 0.05) among treatments according to Tukey’s test for the given day.

The activities of jasmine flower PAL, PPO, and POD enzymes in treated and untreated flowers over the course of storage are shown in **Figure 6**. The initial PAL activity was 1915.73 U/g/h, and values increased in all samples on day 3. Thereafter, PAL activity in the control changed only slightly, while in treated flowers it decreased rapidly. On days 9 and 12, the untreated samples showed significantly higher PAL activity than JEN-treated flowers. After 12 days of storage, the final PAL activity of jasmine flowers treated with 0.5, 1, and 2 mg/mL JEN was 1116.33, 1187.75, and 1183.7 U/g/h, respectively, whereas the activity in the control was 1947.4 U/g/h (**Figure 6A**).

The initial PPO activity in jasmine flowers was 39.24 U/g min. In control samples, this activity first increased, reached a peak, and then slightly decreased. In treated samples, PPO activity showed a continuous decrease relative to the control. After 12 days of storage, the PPO activity of flowers treated with 0.5, 1, and 2 mg/mL JEN was 90.60, 82.05 and 86.20 U/g/min, respectively, whereas that in the control was 129.86 U/g/min (**Figure 6B**).

POD activity was increased in all flowers on day 3, maintained for a period, and then decreased rapidly. On days 3, 6, and 9, POD activity in untreated samples was comparable to those treated with JEN. After 12 days of storage, JEN application was observed to significantly reduce POD activity (**Figure 6C**).

##### 3.4.2.6 Observation by scanning electron microscopy

**Figure 7:**
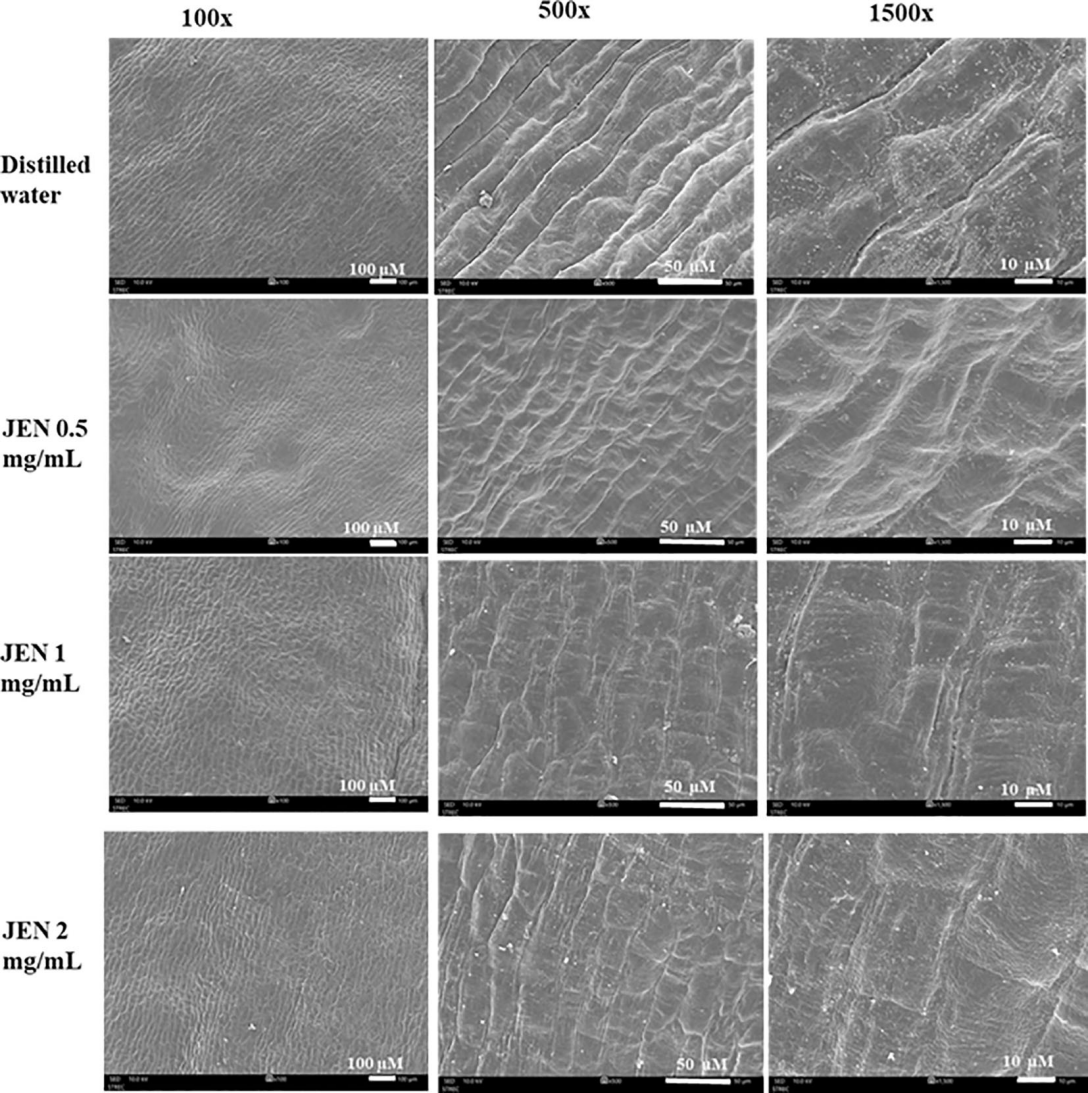
Scanning electron micrographs of the adaxial epidermis of petal tissues of jasmine flowers treated with *J. officinale* nanoemulsion on day 6 of storage. Magnification 100×, scale bar = 100 μM; 500×, scale bar = 50 μM; 1500×, scale bar = 10 μM.

Scanning electron micrographs on day 6 revealed the jasmine petals to have epidermal cells of the tabular rugose type, with longitudinal striations on the adaxial surface. Petals of control flowers showed cell wall breaking and shrinkage in the adaxial epidermis cells and those treated with 0.5 mg/mL JEN initiated shrinkage in the adaxial epidermis. Conversely, jasmine flowers treated with 2 mg/mL JEN exhibited a smooth petal surface free of cell wall breakdown or shrinkage (**Figure 7**).

## 4 Discussion

This work highlighted an innovative postharvest treatment using a plant essential oil to delay the appearance of brown pigment on jasmine petals. In the present study, *J. officinale* essential oil demonstrated the strongest suppression of POD activity in jasmine flowers (**Table 1**), and hence was selected for *in vivo* application to jasmine flowers along with assessment of its chemical composition and antioxidant properties. Benzyl alcohol and benzyl acetate were found to be the most abundant compounds (**Table 2**). The chemical composition of *J. officinale* essential oil has previously been reported by many authors. Jirovetz et al. (2007) similarly identified benzyl acetate (23.7%) and benzyl benzoate (20.7%) as the major constituents. Meanwhile, Muttiah et al. (2019) reported 3,7,11,15-tetramethyl-2-hexadecen-1-ol (12.31%) and benzyl acetate (11.50%) as the major constituents. The high concentration of aromatic compounds, particularly benzyl alcohol, linalool, and their derivatives, suggests this essential oil to possess floral-flowery as well as fruity odor, and to have the strong floral and sweet aromatic characteristics typical of jasmine scents (Upadhyay and Holbrook, 2004; Issa et al., 2020).

Incorporation of essential oils into aqueous matrices is somewhat constrained by their poor solubility in water (Yammine et al., 2024). Nanoemulsions, characterized by a droplet radius less than 100 nm, have been demonstrated to improve the bioavailability, bioaccessibility, and solubility of hydrophobic substances, owing to their increased specific surface area (Doost et al., 2019). Accordingly, JEN was formed as an aqueous solution containing 3 g of *J. officinale* essential oil and 1 g of a mixture of Tween 80 (surfactant) and Span 85 (co-surfactant) in a weight ratio of 1:4, all combined under magnetic stirring. This spontaneous or self-emulsification method (a low-energy emulsification technique) successfully produced a nanoemulsion with droplet sizes in the nano-range (**Table 3**). Nanoemulsion production by this method relies on the viscosity, interfacial tension, and polarity of the oil phase along with the selection of both an appropriate solvent and an appropriate emulsifier. When the surfactant alone is not sufficient to decrease surface tension, the necessary reduction can be achieved by pairing it with a co-surfactant (Mushtaq et al., 2023). There are several reports of spontaneous emulsification generating nanoemulsions (Lima et al., 2021; Tan et al., 2023). When characterizing an emulsion, the PDI reflects the width of the particle size distribution; a perfectly uniform sample would have a PDI of 0.0 (Kale and Deore, 2016). For JEN, the recorded PDI value was 0.191, and the particle size distribution had a narrower range than in the produced microemulsions (**Table 3**). A PDI value of less than 0.2 indicates relative homogeneity of a nanoemulsion (Shakeel et al., 2007).

During storage at 10 ± 3 °CC for 12 days, all flowers demonstrating increasing fresh weight loss as storage time increased, indicating that JEN did not prevent fresh weight loss (**Figure 4A**). Jasmin flowers naturally have a typical climacteric respiratory rise (Isac et al., 2016) that leads to intense respiratory activity and water loss. Likewise, some browning occurred in all flower samples. The PAL activity showed an initial increase, peaked, and then decreased (**Figure 6A**), and was related to the browning as increased PAL activity facilitates *de novo* biosynthesis of phenolic compounds, a process also documented in cut and peeled vegetables and fruit (Song et al., 2019; Xiao et al., 2022). Control flowers exhibited substantial increases of PAL and PPO activity, which led to higher phenol accumulation. Phenols are subsequently oxidized by PPO, which leads to browning (Singh et al., 2018). This petal discoloration during storage was alleviated by treatment with JEN, as evidenced by the comparatively delayed decreases in color retention score (**Figure 4**) and the maintenance of low levels of PAL and PPO activities (**Figure 6**). This phenomenon suggests that the essential oil successfully delayed accumulation of phenolic compounds and limited their conversion to brown pigment by PPO. All JEN-treated samples exhibited lower POD activities than controls at the end of storage (**Figure 6C**). POD is a crucial oxyradical detoxification enzyme that operates on multiple substrates and catalyzes several reactions, variously resulting in browning, discoloration, off-flavors, and nutritional degradation (Alikhani-Koupaei et al., 2014). The lower activity of POD in flowers treated with JEN therefore resulted in alleviated deterioration.

In the *in vitro* study, POD in jasmine flowers was inhibited by treatment with flower essential oils, particularly the *J. officinale* essential oil. Bioactive compounds that are present in essential oils act to reduce the activity of peroxidases, including POD. Ponce et al. (2004) similarly claimed that the antioxidant properties of essential oils can lead to peroxidase activity reduction in leafy vegetables. The *in vivo* study in this work additionally demonstrated that treatment with JEN suppressed the activity of PAL, PPO, and POD. In addition to the oil’s reducing properties, it presumably contains chemical constituents that act as competitive inhibitors of the three enzymes. Chen et al. (2017) observed eugenol to have inhibitory effects on PAL, PPO, and POD activity in lettuce, with the inhibition kinetics indicating it to be a competitive inhibitor of these enzymes. *J. officinale* essential oil is reported to scavenge DPPH• radicals through an electron or hydrogen donation mechanism (Gulcin and Alwasel, 2023), and to reduce ferric ions based on electron transfer rather than hydrogen atom transfer (Prior et al., 2005), which probably contribute to its anti-browning activity (Sae-leaw and Benjakul, 2019). PPO is a dicopper-containing oxidase that harbors Cu^2+^ in its active site. The browning process is the result of this Cu^2+^ interacting with the substrate and oxygen (Sae-Leaw and Benjakul, 2019). *J. officinale* essential oil has demonstrated metal chelating capacity (**Table 2**), which allows for the sequestration of copper atoms that would otherwise occupy the PPO active site, thereby impeding PPO catalytic activity; this action is similar to that of natural antioxidants such as quercetin (Wong et al., 2021). Quercetin has been reported to be effective in controlling browning and maintaining the quality of fresh-cut potatoes (Kasnak, 2022). Among the JEN treatments tested in this study, 2 mg/mL JEN resulted in both the highest freshness index and the least bud opening. This effect may result from the abovementioned antioxidant responses, potentially linked to a delayed senescence that reduces ethylene biosynthesis. Most essential oils function as ethylene antagonists, apparently disrupting ethylene synthesis (Sisler et al., 2006). The SEM observation in this study on day 6 revealed the adaxial epidermal cells of petals in control and low-JEN-treated jasmine flowers to be shrunken and collapsed, with intercellular spaces apparent (**Figure 7**). These anatomical changes indicate that flowers in both treatments experienced senescence (O’ Donoghue, 2006). Meanwhile, petals from flowers treated with higher concentrations of JEN exhibited delayed cell wall collapse, which is beneficial for maintaining cell compartmentation and avoiding the enzyme-substrate interactions that result in petal browning (Lim and Wong, 2018). The results indicated that JEN served as an effective delivery vehicle for bioactive chemicals, facilitating bioactive compounds with targeted delivery and better action. The browning inhibition of jasmine flowers with *J. officinale* essential oil and its constituents was achieved by reducing PPO, POD, and PAL activities, or by directly or indirectly sequestering harmful free radicals.

## 5 Conclusion

This work highlights an innovative protocol to prevent tissue browning using flower essential oil. The *in vitro* study determined the essential oil of *J. officinale* to have the most inhibitory effect on POD activity, highlighting it as a candidate alternative anti-browning agent for the preservation of jasmine flowers *in vivo*. GC-MS analysis indicated the *J. officinale* essential oil to be dominated by benzyl alcohol (20.68%) and benzyl acetate (19.87%). A rapid, easy and reproducible method has been developed for the preparation of *J. officinale* essential oil in nanoemulsion with oil droplet size of 70.2 ± 0.39 nm using a spontaneous emulsification. Due to its small-size droplets, nanoemulsion can function as a delivery vehicle with a targeted mechanism of action. The subsequent *in vivo* study showed treatment with 1 and 2 mg/mL JEN to be effective in controlling browning and maintaining the quality of jasmine flowers, achieving a minimum flower opening index, high freshness score, and high color retention index after 12 days of storage. This mechanism for this change due to the essential oil demonstrated DPPH radical scavenging, iron chelating, and reducing capabilities, indicating that it may function as an antioxidant, reducing agent, chelating agent, or combination thereof to inhibit enzymatic browning. In addition, essential oil significantly reduced PAL and PPO activity, preventing accumulation of the phenolic compounds that are substrates of PPO. Likewise, POD activity was reduced with oil treatment. With respect to SEM observation, jasmine flower epidermis cells showed treatment with JEN to delay cell wall collapse and preserve cell compartmentation, indicating postponement of the browning process.

## Data Availability

The original contributions presented in the study are included in the article/supplementary material. Further inquiries can be directed to the corresponding author.

## References

[B1] Aghdam M. S. Jannatizadeh A. Nojadeh M. S. Ebrahimzadeh A. (2019). Exogenous melatonin ameliorates chilling injury in cut anthurium flowers during low temperature storage. Postharvest Biol. Technol. 148, 184–191. doi: 10.1016/j.postharvbio.2018.11.008

[B2] Ali S. M. Nidoni U. Palanimuthu S. H. Ramappa K. Ramesh G. Naik N. (2024). Enhancing shelf life and freshness retention in jasmine flowers (*Jasminum multiflorum* L.) through post-harvest chemical treatments. Int. J. Advanced Biochem. Res. 8, 265–270. doi: 10.33545/26174693.2024.v8.i3Sd.720

[B3] Alikhani-Koupaei M. Mazlumzadeh M. Sharifani M. Adibian M. (2014). Enhancing stability of essential oils by microencapsulation for preservation of button mushroom during postharvest. Food Sci. Nutr. 2, 526–533. doi: 10.1002/fsn3.129 25473510 PMC4237482

[B4] Arteaga J. F. Ruiz-Montoya M. Palma A. Alonso-Garrido G. Pintado-S.-and-Rodríguez-Mellado J. M. (2012). Comparison of the simple cyclic voltammetry (CV) and DPPH assays for the determination of antioxidant capacity of active principles. Molecules 17, 5, 5126 5138. doi: 10.3390/molecules17055126 22555300 PMC6268035

[B5] Boonprasert J. Chaiprasart P. Krajayklang M. (2018). The effect of boric acid on physical quality change in Jasmine (*Jasminum sambac*) flowers after harvest. Agric. Sci. J. 49, 111–114.

[B6] Chen X. Ren L. Li M. Qian J. Fan J. Du B. (2017). Effects of clove essential oil and eugenol on quality and browning control of fresh-cut lettuce. Food Chem. 214, 432–439. doi: 10.1016/j.foodchem.2016.07.101 27507495

[B7] Cortés H. Hernández-Parra H. Bernal-Chávez S. A. Prado-Audelo M. L. D. Caballero-Florán I. H. Borbolla-Jiménez F. V.. (2021). Non-ionic surfactants for stabilization of polymeric nanoparticles for biomedical uses. Materials 14, 3197. doi: 10.3390/ma14123197 34200640 PMC8226872

[B8] Doost S. A. Kassozi V. Grootaert C. Claeys M. Dewettinck K. Camp V.. (2019). Self-assembly, functionality, and *in-vitro* properties of quercetin loaded nanoparticles based on shellac-almond gum biological macromolecules. Int. J. Biol. Macromolecules 129, 1024–1033. doi: 10.1016/j.ijbiomac.2019.02.071 30794898

[B9] Gao M. Feng L. Jiang T. (2014). Browning inhibition and quality preservation of button mushroom (*Agaricus bisporus*) by essential oils fumigation treatment. Food Chem. 149, 107–113. doi: 10.1016/j.foodchem.2013.10.073 24295683

[B10] Golrokh H. K. Somayeh R. (2023). [amp]]gamma;-Aminobutyric acid (GABA) inhibits the enzymatic browning of cut Narcissus tazetta cv. ‘Shahla-e-Shiraz’ flowers during vase life. J. Plant Growth Regul. 42, 2602–2612. doi: 10.1007/s00344-022-10730-1

[B11] Gulcin I. Alwasel S. H. (2023). DPPH radical scavenging assay. Processes 11, 2248. doi: 10.3390/pr11082248

[B12] Huang S.-J. Lin S.-Y. Wang T.-T. Hsu F.-C. (2020). Combining acetic acid and ethanol as an anti-browning treatment for lettuce butt discoloration through repression of the activity and expression of phenylalanine ammonia lyase. Postharvest Biol. Technol. 164, 111151. doi: 10.1016/j.postharvbio.2020.111151

[B13] Isac X. A. Rajadurai K. Jawaharlal M. Selvan K. Uma D. Roy H.. (2016). Aroma profiling of jasmine (*Jasminum sambac* Ait.) flowers using electronic nose technology. J. Appl. Horticulture 18, 19–24. doi: 10.37855/jah.2016.v18i01.05

[B14] Issa M. Y. Mohsen E. Younis I. Y. Nofal E. S. Farag M. A. (2020). Volatiles distribution in jasmine flowers taxa grown in Egypt and its commercial products as analyzed via solid-phase microextraction (SPME) coupled to chemometrics. Ind. Crops Products 144, 112002. doi: 10.1016/j.indcrop.2019.112002

[B15] Jirovetz L. Buchbauer G. Schweiger T. Denkova Z. Slavchev A. Stoyanova A.. (2007). Chemical composition, olfactory evaluation and antimicrobial activities of *Jasminum grandiflorum* L. absolute from India. Natural Product Commun. 2, 407-412. doi: 10.1177/1934578x0700200411

[B16] Kale S. Deore S. (2016). Emulsion micro emulsion and nano emulsion: A review. Systematic Rev. Pharm. 8, 39–47. doi: 10.5530/srp.2017.1.8

[B17] Kasnak C. (2022). Evaluation of the anti-browning effect of quercetin on cut potatoes during storage. Food Packaging Shelf Life 31, 100816. doi: 10.1016/j.fpsl.2022.100816

[B18] Lavanya V. Nidoni U. Kurubar A. Sharanagouda H. Ramachandra C. (2016). Effect of pre-treatment and different packaging materials on shelf-life of jasmine flowers (*Jasminum sambac*). Environ. Ecol. 34, 341–345.

[B19] Lim W. Y. Wong C. W. (2018). Inhibitory effect of chemical and natural anti-browning agents on polyphenol oxidase from ginger (*Zingiber officinale* Roscoe). J. Food Sci. Technol. 55, 3001–3007. doi: 10.1007/s13197-018-3218-7 30065409 PMC6045987

[B20] Lima T. S. Silva M. F. S. Nunes X. P. Colombo A. V. Oliveira H. P. Goto P. L.. (2021). Cineole-containing nanoemulsion: Development, stability, and antibacterial activity. Chem. Phys. Lipids 239, 105113. doi: 10.1016/j.chemphyslip.2021.105113 34216586

[B21] Lou Z. Chen J. Yu F. Wang H. Kou X. Ma C.. (2017). The antioxidant, antibacterial, antibiofilm activity of essential oil from *Citrus medica* L. Var. sarcodactylis its nanoemulsion. LWT 80, 371–377. doi: 10.1016/j.lwt.2017.02.037

[B22] Mushtaq A. Mohd Wani S. Malik A. R. Gull A. Ramniwas S. Ahmad Nayik G.. (2023). Recent insights into Nanoemulsions: Their preparation, properties and applications. Food Chemistry: X 18, 100684. doi: 10.1016/j.fochx.2023.100684 37131847 PMC10149285

[B23] Muttiah N. N. Lim V. Saidin A. (2019). Chemical composition and synergistic repellent activity of *Jasminum officinale* and *Anthemis nobilis* essential oils against *Aedes aegypti* mosquitoes. Malays. J. Med. Health Sci. 15 (Supp.9), 30–36.

[B24] O’ Donoghue E. (2006). Flower petal cell walls: Changes associated with flower opening and senescence. New Z. J. Forestry Sci. 36, 130–144.

[B25] Piva G. Fracassetti D. Tirelli A. Mascheroni E. Musatti A. Inglese P.. (2017). Evaluation of the antioxidant/antimicrobial performance of *Posidonia oceanica* in comparison with three commercial natural extracts and as a treatment on fresh-cut peaches (*Prunus persica* Batsch). Postharvest Biol. Technol. ,124, 54–61. doi: 10.1016/j.postharvbio.2016.10.001

[B26] Ponce A. G. Del Valle C. E. Roura S. I. (2004). Natural essential oils as reducing agents of peroxidase activity in leafy vegetabls. LWT - Food Sci. Technol. 37, 199–204. doi: 10.1016/j.lwt.2003.07.005

[B27] Prior R. L. Wu X. Schaich K. (2005). Standardized methods for the determination of antioxidant capacity and phenolics in foods and dietary supplements. J. Agric. Food Chem. 53, 4290–4302. doi: 10.1021/jf0502698 15884874

[B28] Sae-Leaw T. Benjakul S. (2019). Prevention of melanosis in crustaceans by plant polyphenols: A review. Trends Food Sci. Technol. 85, 1–9. doi: 10.1016/j.tifs.2018.12.003

[B29] Salvia-Trujillo L. Rojas-Graü A. Soliva-Fortuny R. Martín-Belloso O. (2015). Physicochemical characterization and antimicrobial activity of food-grade emulsions and nanoemulsions incorporating essential oils. Food Hydrocolloids 43, 547–556. doi: 10.1016/j.foodhyd.2014.07.012

[B30] Seyyed J. M. Shahram S. (2011). Peroxidase activity in response to applying natural antioxidant of essential oils in some leafy vegetabls. Aust. J. Crop Sci. 5, 494–499.

[B31] Shakeel F. Baboota S. Ahuja A. Ali J. Aqil M. Shafiq S. (2007). Nanoemulsions as vehicles for transdermal delivery of aceclofenac. AAPS PharmSciTech 8, 104. doi: 10.1208/pt0804104 PMC2750690 18181525

[B32] Singh N. Rajini P. S. (2004). Free radical scavenging activity of an aqueous extract of potato peel. Food Chem. 85, 611–616. doi: 10.1016/j.foodchem.2003.07.003

[B33] Singh B. Suri K. Shevkani K. Kaur A. Kaur A. Singh N. (2018). “Enzymatic browning of fruit and vegetabls: A review,” in Enzymes in food technology: improvements and innovations, Singapore: Springer, 63–78.

[B34] Sisler E. C. Grichko V. P. Serek M. (2006). Interaction of ethylene and other compounds with the ethylene receptor: agonists and antagonists (Berlin, Heidelberg, Germany: Springer).

[B35] Song M. Wu S. Shuai L. Duan Z. Chen Z. Shang F.. (2019). Effects of exogenous ascorbic acid and ferulic acid on the yellowing of fresh-cut Chinese water chestnut. Postharvest Biol. Technol. 148, 15–21. doi: 10.1016/j.postharvbio.2018.10.005

[B36] Sriwichan T. Chutichudet B. Chutichudet P. (2016). Effects of calcium carbonate on browning appearance in jasmine flower. J. Sci. Technol. Mahasarakham Univ. 36, 341–347.

[B37] Sundararajan B. Moola A. K. Vivek K. Kumari B. D. R. (2018). Formulation of nanoemulsion from leaves essential oil of *Ocimum basilicum* L. and its antibacterial, antioxidant and larvicidal activities (*Culex quinquefasciatus*). Microbial Pathogenesis 125, 475–485. doi: 10.1016/j.micpath.2018.10.017 30340015

[B38] Tan T. N. Mnocaran Y. P. A. P. Ramli M. E. Utra U. Ariffin F. Yussof N. S. (2023). Physical properties, antioxidant activity, and *in vitro* digestibility of essential oil nanoemulsions of Betel and Pandan leaves. ACS Food Sci. Technol. 3, 150–160. doi: 10.1021/acsfoodscitech.2c00296

[B39] Thaipong K. Boonprakob U. Crosby K. Cisneros-Zevallos L. Byrne D. H. (2006). Comparison of ABTS, DPPH, FRAP, and ORAC assays for estimating antioxidant activity from guava fruit extracts. J. Food Composition Analysis. 19, 669–675. doi: 10.1016/j.jfca.2006.01.003

[B40] Upadhyay U. D. Holbrook E. H. (2004). Olfactory loss as a result of toxic exposure. Otolaryngologic Clinics North America 37, 1185–1207. doi: 10.1016/j.otc.2004.05.003 15563910

[B41] Wang B. Wang Y. Huang Y. Jiang Y. He J. Xiao Y. (2022). Anti-browning effects of citronellal on fresh-cut taro (*Colocasia esculenta*) slices under cold storage condition. Front. Sustain. Food Syst. 6. doi: 10.3389/fsufs.2022.1001362

[B42] Wong C. W. Yen Fang Toh A. Lim W. Y. (2021). Effect of heated onion extract on white button mushroom (*Agaricus bisporus*) polyphenol oxidase. Carpathian J. Food Sci. Technol. 13, 16–23. doi: 10.34302/crpjfst/2021.13.4.2

[B43] Xiao Y. Zhang J. Jiang Y. Yuan Y. Xie J. He J.. (2022). Cinnamic acid treatment reduces the surface browning of fresh-cut taro. Scientia Hortic. 291, 110613. doi: 10.1016/j.scienta.2021.110613

[B44] Xu J. Zhou L. Miao J. Yu W. Zou L. Zhou W.. (2020). Effect of cinnamon essential oil nanoemulsion combined with ascorbic acid on enzymatic browning of cloudy apple juice. Food Bioprocess Technol. 13, 860–870. doi: 10.1007/s11947-020-02443-8

[B45] Yammine J. Chihib N.-E. Gharsallaoui A. Ismail A. Karam L. (2024). Advances in essential oils encapsulation: development, characterization and release mechanisms. Polymer Bull. 81, 3837–3882. doi: 10.1007/s00289-023-04916-0

